# The protective effect of hydroxyethyl starch solution on the glycocalyx layer in an acute hemorrhage mouse model

**DOI:** 10.1007/s00540-019-02692-8

**Published:** 2019-10-15

**Authors:** Kohji Uzawa, Akira Ushiyama, Shingo Mitsuda, Tadao Ando, Marie Sawa, Hideki Miyao, Tomoko Yorozu

**Affiliations:** 1 Department of Anesthesiology, Kyorin University School of Medicine, 6-20-2 Shinkawa, Mitaka-shi, Tokyo, 181-8611 Japan; 2 Department of Environmental Health, National Institute of Public Health, 2-3-6 Minami, Wako, Saitama 351-0197 Japan; 3 Meiji Pharmaceutical University Graduate School of Pharmaceutical Sciences, 2-522-1, Noshio, Kiyose-shi, Tokyo, 204-8588 Japan; 4 Department of Anesthesiology, Saitama Medical Center, Saitama Medical University, 1981, Kamoda, Kwagoe, Saitama 350-8550 Japan

**Keywords:** Glycocalyx layer, Hydroxyethyl starch solution, Dorsal skin chamber, Microvascular circulation, Fluid resuscitation

## Abstract

**Purpose:**

Fluid therapy focused on glycocalyx (GCX) protection in hemorrhagic shock is a current focus of research. Hydroxyethyl starch (HES) solution is commonly used for fluid resuscitation; however, its effects on the GCX remain unclear. The primary aim of this study was to explore the protective effect of HES130 in maintaining GCX thickness and reducing plasma syndecan-1 expression.

**Methods:**

An acute hemorrhage murine model with the dorsal skin chambers was used to measure GCX thickness and to evaluate vascular permeability. Groups of mice were treated with normal saline (NS), albumin (NS-A), HES130 (NS-V), or no exsanguination or infusion (C). We measured syndecan-1 plasma concentrations, performed blood gas analysis, and analyzed the 7-day cumulative mortality.

**Results:**

GCX thickness in NS mice was significantly reduced compared to that in group C, but no other groups showed a difference compared to group C. The plasma concentration of syndecan-1 was significantly higher in NS mice than in group C. There were no significant differences in the fluorescence intensity of dextran in the interstitial space. HES70 leakage was suppressed in NS-V mice compared to those in other groups. HES70 was localized to the inner vessel wall in C, NS, and NS-A mice, but not in group NS-V. Blood gas analysis indicated that pH and lactate showed the greatest improvements in NS-V mice. The 7-day cumulative mortality rate was the highest in group NS.

**Conclusion:**

Resuscitation with HES130 protected the GCX and suppressed vascular permeability of HES70 during early stages of acute massive hemorrhage.

**Electronic supplementary material:**

The online version of this article (10.1007/s00540-019-02692-8) contains supplementary material, which is available to authorized users.

## Introduction

In clinical practice, it is crucial to select the optimal fluid resuscitation strategy for patients with emergency massive hemorrhage requiring urgent blood transfusions. The shock caused by massive hemorrhage is known to impair endothelial function and induce hyperpermeability [1], leading to a poor prognosis. Fluid replacement with blood products, colloids, and crystalloids affects morbidity and mortality in acute hemorrhage patients. The fluid management of patients with hemorrhagic shock is an important concern for anesthesiologists.

In recent years, glycocalyx (GCX), which is located on the luminal surface of the endothelial cells, has been a research topic in terms of fluid management because it plays an important role in maintaining vascular wall integrity and preventing plasma leakage [2]. Nevertheless, the mechanisms controlling the effects of artificial colloid administration on the GCX are not fully understood.

Under severe disease conditions, the GCX may collapse owing to various factors [2–4]. In particular, the onset of massive hemorrhaging may occur, a phenomenon known as shock-induced endotheliopathy [1, 5]. In addition, the increased expression of disintegration markers in the GCX has been reported to be associated with increased mortality in trauma patients [6]. It was reported that syndecan-1 and hyaluronic acid, important components of the GCX, are released into the blood of patients with severe trauma due to increased hyperpermeability and low plasma colloid osmotic pressure [7, 8].

Therefore, preventing GCX degradation is important when administering fluid therapy to patients with acute hemorrhage. In patients with massive hemorrhages, although fresh frozen plasma (FFP) was found to be a highly effective treatment to suppress GCX degradation [9, 10], these transfusion products are not always readily available [11, 12]. On the other hand, artificial colloid solutions can be rapidly administered, and are more effective in sustaining blood pressure than crystalloid solutions [13] because colloid fluid loading maintains colloid osmotic pressure and decreases inflammation [14]. However, its effects on the GCX have not been clarified.

Several fluid therapy studies have focused on the endothelial surface layer (ESL) and GCX in hemorrhagic shock [11, 12] by comparing the effects of physiological saline, Ringer's solution, albumin, and FFP. However, hydroxyethyl starch (HES) solutions have not been investigated for their role in microcirculation. Therefore, we investigated the effectiveness of HES in protecting the GCX during acute hemorrhagic shock. This study aimed to clarify the influence of HES:130/0.4/9 (HES130) on GCX under severe hemorrhagic conditions of a mouse model, using intravital microscopy [15] with fluorescent-labeled lectin on mouse dorsal skinfold chambers (DSC) to directly visualize the endothelial GCX [16] and evaluate changes in vascular permeability in vivo.

We predicted that HES130 would attenuate GCX injury during early stages of acute massive hemorrhage and thus inhibit vascular permeability. The primary aim of this study was to evaluate the changes in GCX thickness and blood syndecan-1 concentrations in a mouse model. The secondary aim was to characterize the leakage of tetramethylrhodamine (TMR)-labeled dextran (molecular weight, 40 kDa; DEX40) and fluorescein isothiocyanate (FITC)-labeled HES (molecular weight, 70 kDa; HES70) into the interstitial space. We also performed blood gas analysis (BGA) and investigated the 7-day cumulative mortality.

## Methods

### Animals

**Fig. 1:**
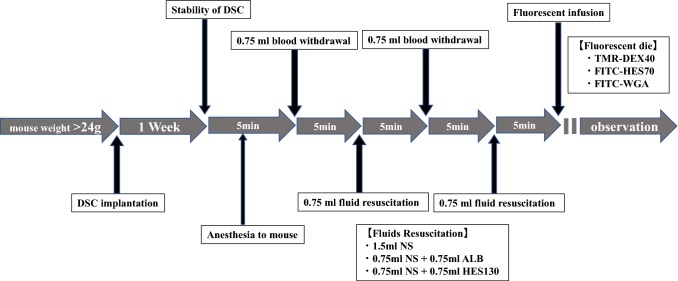
Experimental protocol. A dorsal skinfold chamber (DSC) was surgically attached to male BALB/c mice under anesthesia. At least 1 week after DSC attachment, the mice were anesthetized again, and the acute bleeding experiment was started as follows. First, 0.75 ml blood was withdrawn via the jugular vein at a rate of 0.1 ml per 3 s. After 5 min, the same amount of the allocated fluid in each group was administered. This procedure was then repeated for a total blood loss of 1.5 ml with 1.5 ml fluid resuscitation. After 5 min, mice were injected with tetramethyl rhodamine-labeled dextran (TMR-DEX40) and fluorescein isothiocyanate-labeled hydroxyethyl starch (FITC-HES70), and their fluorescence intensities were measured at regular intervals from 5 to 90 min. In separate groups of mice treated using the same protocol, fluorescein isothiocyanate-labeled wheat germ agglutinin (FITC-WGA) was administered in place of TMR-DEX40 and FITC-HES70 to observe changes in glycocalyx (GCX) thickness at 30 min after administration. NS, normal saline; ALB, albumin; HES130, hydroxyethyl starch 130

Male BALB/c mice were purchased from Japan SLC, Inc. (Shizuoka, Japan). Mice were fed a standard pellet diet (FR-2; Funabashi Farm Co., Chiba, Japan), provided with tap water acidified with hydrochloric acid ad libitum, and housed in individually ventilated cage systems (Super Mouse 1400TM Micro-Isolator Rack; Lab Products, Inc., Seaford, DE, USA) with a 12-h light/dark cycle. All experimental protocols (Fig. 1) were approved by the Committee for Animal Experiments at the National Institute of Public Health (protocol number 30–006) and were in accordance with the guidelines and laws for animal experiments in Japan.

### Chemicals and reagents

Albumin was purified from mouse serum (Kohsin Bio Co. Ltd., Saitama, Japan) according to the standard method [17] with some modifications. Briefly, 200 ml mouse serum was filtered through a 0.45-μm mesh filter to remove debris. The pH of the serum was adjusted to 7.2 with 1 M acetic acid and gradually cooled to − 5 °C. Ethanol (95% *w*/*v*) at − 30 °C was slowly added to a final concentration of 19% ethanol, and the solution was incubated overnight at − 5 °C. The serum was then centrifuged for 5 min at 3000×*g* and 5 °C, and the supernatant was collected. The supernatant was adjusted to pH 5.2 with acetate buffer (pH 4.0, containing 0.8 M sodium acetate and 4 M acetic acid) with constant stirring at − 5 °C, reducing the ethanol concentration to 18%. The sample was incubated overnight at − 5 °C and then centrifuged for 5 min at 3000×*g* and 5 °C. The supernatant was collected and placed in an ion exchange chromatography column packed with diethylaminoethanol (DEAE)–sepharose (HiTrap DEAE FF; GE Healthcare, Chicago, IL, USA) equilibrated with 20 mM acetate buffer (pH 5.2) at 18 °C. Albumin was eluted from the column using 25 mM acetate buffer (pH 4.5), neutralized to pH 7.2, and concentrated with a centrifugal filter unit with a nominal molecular weight limit of 50 kDa (Amicon Ultra-15 Centrifugal Filter Units; Merck, Darmstadt, Germany). The purity of the albumin was validated by SDS-PAGE. Protein concentration was determined using the Bradford Ultra Total Protein Quantitation Kit (Expedeon Inc., San Diego, CA, USA), and adjusted to 5% (*w*/*v*) for administration in mice. The albumin solution was kept at − 30 °C until use.

HES powder (70/0.55/4; mean molecular weight: 70 kDa) was obtained from Otsuka Pharmaceutical Factory, Inc. (Naruto, Japan). FITC-conjugated HES was prepared as previously described [17, 18]. Briefly, 1 ml anhydrous dimethyl sulfoxide (DMSO), 2 ml pyridines (Wako Pure Chemical Co., Tokyo, Japan), and 2 ml dibutyl tin dilaurate (Sigma Aldrich, St. Louis, MO, USA) were mixed. HES powder (100 mg) was added to the solution, heated to 95 °C until dissolved, and mixed with 10 mg FITC (Isomer I; Sigma Aldrich). Free dye was separated from conjugated HES by ethanol precipitation. The labeled polysaccharide was collected by centrifugation at 1000×*g* for 10 min and the pellet was washed several times in cold ethanol and dried in a vacuum evaporator at room temperature. The final pellet was resuspended in saline, and the low molecular weight fraction (< 3 kDa) was removed using a centrifugal filter (Amicon Ultra-15 Centrifugal Filter Units, Merck); the final solution was kept at − 30 °C until use.

### Dorsal skinfold chamber preparation

DSCs (Supplemental Fig. 1) were used to visualize the microvasculatures. Briefly, the DSC chamber frame was constructed from poly-acetal resin, as in our previous study [15]. Two frames were surgically implanted, so that the extended double layer of the dorsal skin was sandwiched. A coverslip was then fixed with the retaining ring. During the surgical procedure, mice were anesthetized by subcutaneous injection of a mixture of ketamine (90 mg/kg body weight) and xylazine (10 mg/kg body weight). Mice were allowed to acclimatize for at least 1 week before microscopic observation to avoid any inflammatory effects due to surgery.

### Preparation of acute hemorrhage mouse models

Mice were anesthetized by subcutaneous injection of a mixture of ketamine (90 mg/kg body weight) and xylazine (10 mg/kg body weight). To mimic clinical massive hemorrhages, about 60% of the total blood volume (1.5 ml) was removed from the mice. The blood was then replaced at a 1:1 ratio with one of four fluid treatments, as described below.

After inserting a catheter to the common jugular vein, 0.75 ml blood was withdrawn, and 0.75 ml resuscitation fluid was infused. After 5 min, an additional 0.75 ml blood was withdrawn, and 0.75 ml resuscitation fluid was infused. Thereafter, fluorescent dyes (TMR-DEX40 and FITC-HES70) were administered through the catheter to observe the changes in vascular permeability. Next, FITC-labeled wheat germ agglutinin (WGA; Sigma Aldrich) was independently administered through the catheter to measure the GCX thickness index (Fig. 1). BGA was performed 60 min after blood withdrawal. After treatment, the common carotid artery was ligated near the catheter insertion site, the catheter was removed, and the skin was sutured. The 7-day mortality of the treated mice was then measured.

The mice were randomly divided into four groups: control (C), DSC implantation only without blood withdrawal or infusion; saline administration (NS), infusion of 1.5 ml saline after withdrawal of 1.5 ml blood; albumin administration (NS-A), infusion of 0.75 ml saline and 0.75 ml albumin; and HES130 (Voluven; Fresenius Kabi, Bad Homburg, Germany) administration (NS-V), infusion of 0.75 ml saline and 0.75 ml HES130. The details of the mice used for the experiments are shown in Supplementary Table 1.

### Measurement of GCX thickness index after exsanguination

After inducing acute hemorrhaging in the four groups as described above, the mice were left to stabilize for about 5 min and then injected with FITC-WGA. After 30 min, three fluorescent images were obtained in each chamber.

**Fig. 2:**
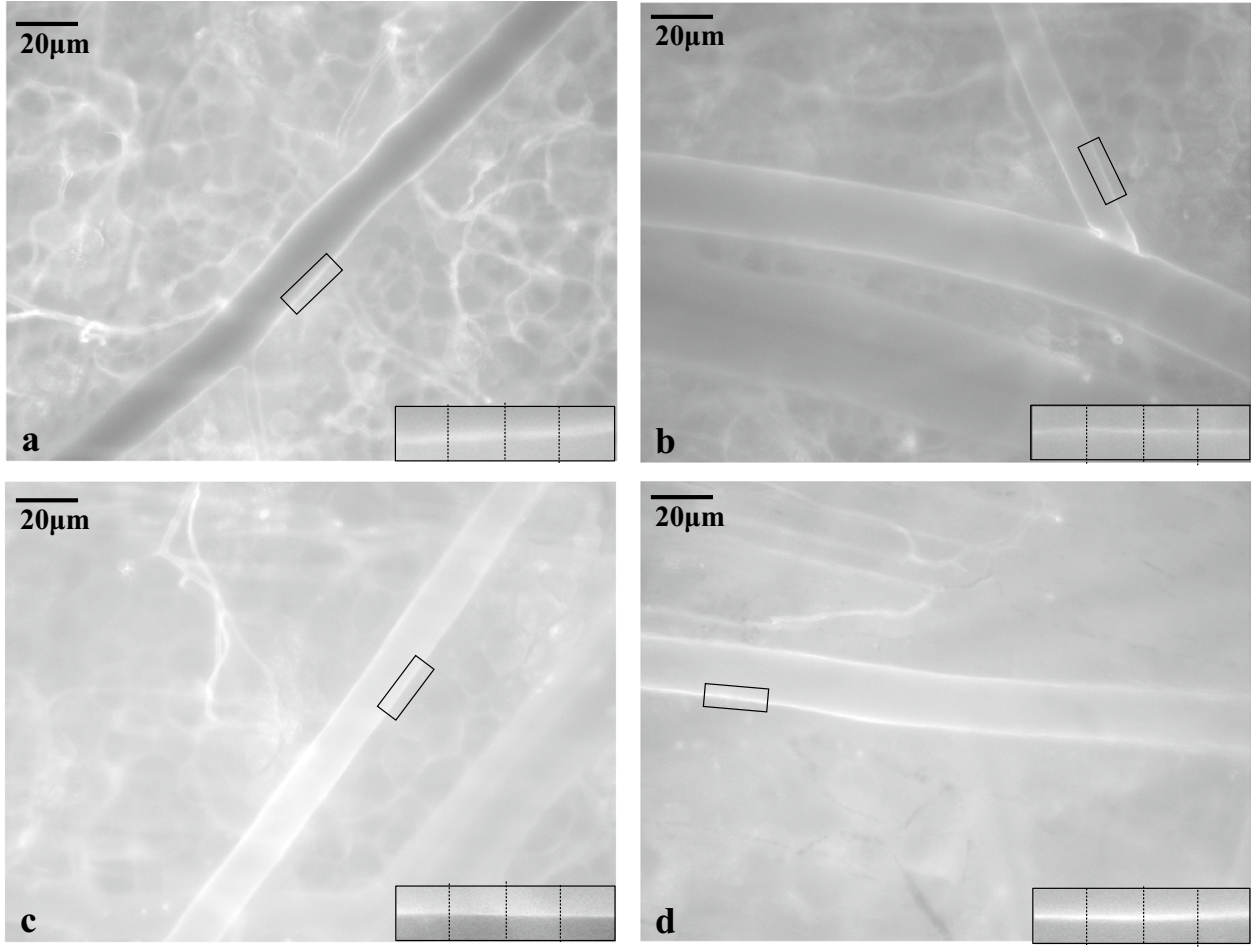
Images of glycocalyx (GCX) stained with FITC-WGA in each group. FITC-WGA was administered after each fluid resuscitation, and then images were obtained 30 min later. The approximately 20 μm artery walls were clearly stained with FITC-WGA lectin. An acute hemorrhage murine model with dorsal skin chambers was used to evaluate GCX thickness index by measuring FITC-WGA lectin. Representative images are shown for each group: no exsanguination or infusion (**a**), administered with 1.5 ml normal saline (**b**), 0.75 ml normal saline + 0.75 ml albumin (**c**), 0.75 ml normal saline + 0.75 ml HES130 (**d**)

The artery walls were clearly illuminated by FITC-WGA-lectin. Fluorescent images of FITC-WGA stained regions were analyzed using ImageJ software. GCX thickness was measured as previously reported [16, 19, 20]. We observed vessels on the two-dimensional surface of the windows. The peripheral vessels exist in a three-dimensional matrix in vivo. When a blood vessel is curved, or runs three-dimensionally across a 2D observation plane, the thickness of the vessel wall will be uneven, and measurements of GCX thickness will be inaccurate. Therefore, we randomly selected and observed three straight sections of blood vessels of approximately 20 μm diameter, which was clearly illuminated with a fluorescent bio-optical microscope. Then, we extracted a partial view of one side of the vessel wall. Three perpendicular lines were drawn on the vessel wall at three points with the same intervals between the ends of the sides of the image (Fig. 2 and Supplemental Fig. 2). We measured the fluorescence intensities on each perpendicular line. We defined the mean values of the fluorescence intensities obtained from nine lines drawn to three arteries as the GCX thickness index of each blood vessel. Then we compared the changes in the GCX thickness index of groups NS, NS-A, and NS-V to that of group C (Fig. 2).

### Measurement of plasma syndecan-1

Blood was removed, resuscitation fluid was added, and the mice were allowed to rest for 60 min; thereafter, blood was collected and the plasma concentration of syndecan-1 was measured using the sCD138 ELISA Kit (Diaclone SAS, Besançon, France) according to the manufacturer’s instructions. Briefly, samples, standards, and diluted biotinylated anti-mouse CD138 antibody were added to precoated wells and incubated for 2 h at 25 °C. After three washes, horseradish peroxidase (HRP)-conjugated streptavidin was added, and the plate was incubated for 1 h at room temperature; the substrate was then added, and the color was allowed to develop for 15–30 min. The absorbance at 450 nm was measured using a microplate reader (Bio-Rad Laboratories, Hercules, CA, USA). The plasma concentration of syndecan-1 was calculated using a standard curve.

### Measurement of vascular permeability after exsanguination

**Fig. 3:**
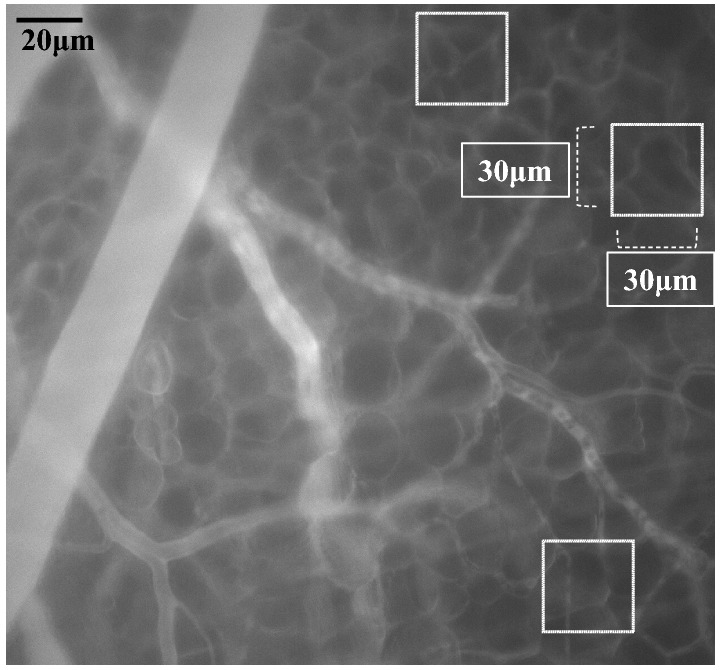
Measurement of fluorescence intensity in the interstitial space. Representative images of the tissue at 60 min after blood removal, HES130 fluid resuscitation, and TMR-DEX40 infusion. Three images were randomly selected. In each image, we randomly selected three squares of interstitial space (30 × 30 μm) containing no vessels

The fluorescence intensity of TMR-DEX40 and FITC-HES70 was measured as an index of leakage into the peripheral area in the DSCs in the four groups. Optimal DSCs were selected for each observation. First, the DSCs with mechanical damage, showing inflammation, without a clearly observable portion of skin, or uneven fluorescence signals due to low peripheral circulation were excluded for the assessment of fluorescence intensities. Second, DSCs with microcirculatory failure were also excluded in the observation. Then, we randomly selected three 30 × 30-μm square areas without any vessels in each DSC window (Fig. 3) to measure the fluorescence intensity in the interstitial space as an index of the leakage of DEX40 and HES70. The mean fluorescence intensity from the three measurements was defined as the fluorescence intensity of the interstitial space in each DSC. We also observed the changes in fluorescence intensity at 5, 15, 30, 60, and 90 min.

ImageJ software was used for the analysis of fluorescence images. The software assigned an integer value to the brightness of the fluorescence using an 8-bit gray scale (range 0–255) for each region of interest. The difference in the value from the first image was used as an index of vascular permeability.

### Measurement of blood gas and 7-day cumulative survival rates

Blood gas measurement was performed in all groups at 60 min after blood withdrawal using the i-STAT 300F Analyzer EC4+ , CG4+ (Abbott, Princeton, NJ, USA).

To measure the survival rate of the mice, acute hemorrhage was induced in a second set of mice using the same treatments described above (*n* = 10 per group; total: *n* = 40). At 2 h after blood removal and infusion, the catheter was removed from the common jugular vein, hemostasis was induced, the wound was sutured, mice were allowed free access to food and drink for 7 days, and then the surviving mice were counted and compared among the four groups.

### Statistical analysis

Statistical tests were carried out using the JMP software package (JMP 14; SAS Inc., Cary, NC, USA). The GCX thickness index and plasma concentration of syndecan-1 were analyzed using one-way analysis of variance (ANOVA) followed by Dunne’s post hoc test, with group C as the reference group. Vascular permeability measurements were analyzed by one-way ANOVA and the Bonferroni post hoc test. A value of *P* < 0.05 was considered statistically significant.

## Results

**Fig. 4:**
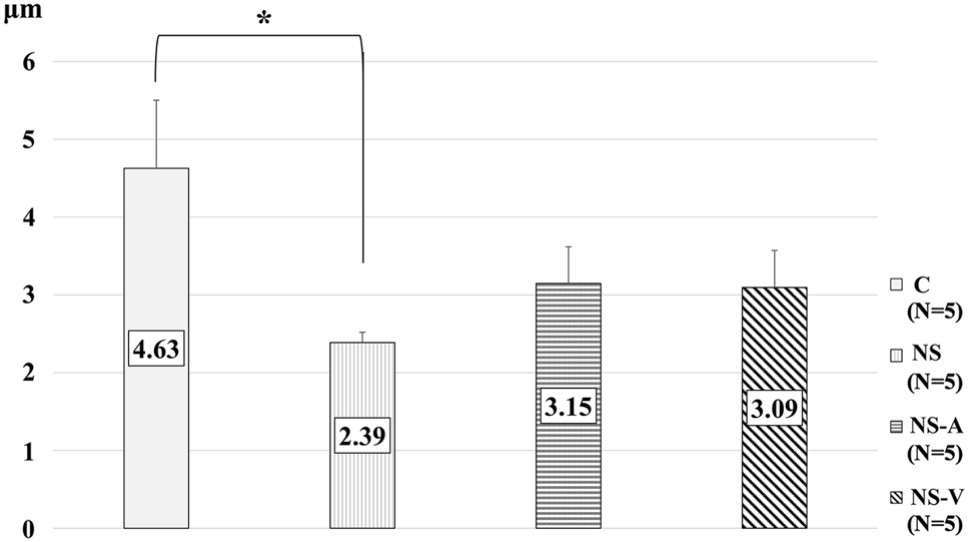
Measurements of the thickness of FITC-WGA -positive layers as a surrogate for glycocalyx (GCX) thickness. The fluorescence intensity of FITC-WGA-positive layers was defined as the GCX index to determine changes in the thickness of the GCX compared to the control group (C). Values are expressed as the mean ± SE; *N* = 5 per group. *C* untreated control group without blood loss or infusion, *NS* normal saline infusion group, *NS-A* normal saline and albumin infusion group, *NS-V* saline and HES130 infusion group. The data were analyzed by one-way analysis of variance (ANOVA) followed by Dunne’s post hoc test. *P < 0.05 compared to C

The average FITC-WGA positive layer thickness in group C was 4.63 ± 1.96 μm, and those in groups NS, NS-A, and NS-V were 2.39 ± 0.30 μm, 3.15 ± 1.06 μm, and 3.09 ± 1.06 μm, respectively. The FITC-WGA-positive layer thickness in group NS was significantly thinner than that in group C (*P* = 0.03). but no other groups (vs. group NS-A, P = 0.183 / vs. group NS-V; *P* = 0.164) showed a significant difference compared to group C (Fig. 4).

**Fig. 5:**
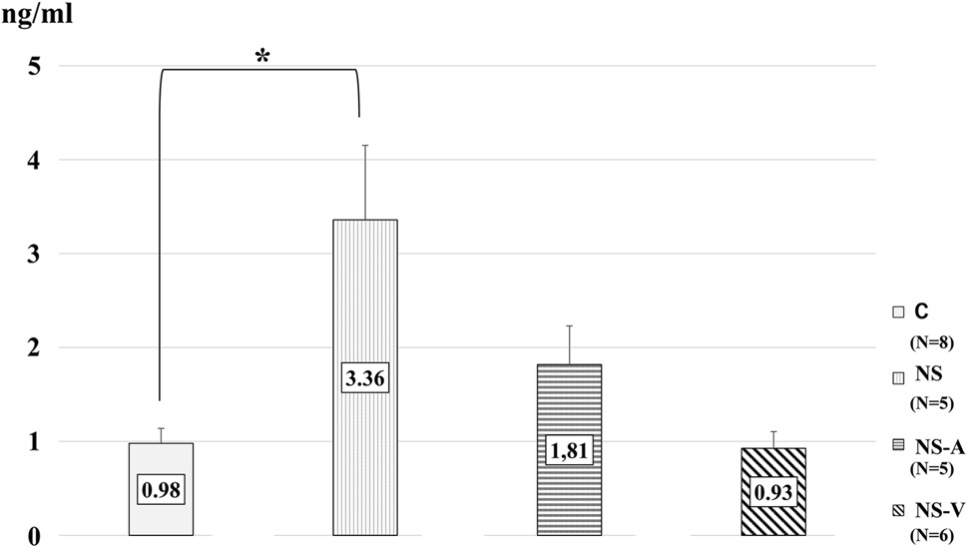
Plasma concentration of syndecan-1. The plasma concentration of syndecan-1 relative to the control group (C) was measured in each group at 60 min after fluid resuscitation. Values are expressed as the mean ± SE. *C* untreated control group without blood loss or infusion (*N* = 8), *NS* normal saline infusion group (*N* = 5), *NS-A* normal saline and albumin infusion group (*N* = 5), *NS-V* saline and HES130 infusion group (*N* = 6). The data were analyzed by one-way analysis of variance (ANOVA) followed by Dunne’s post hoc test. *P < 0.05 compared to C

The average plasma concentration of syndecan-1 in group C at 60 min after blood removal was 0.98 ± 0.45 ng/ml; those in groups NS, NS-A, and NS-V were 3.36 ± 1.78 ng/ml, 1.81 ± 0.92 ng/ml, and 0.93 ± 0.44 ng/ml, respectively. The plasma concentration of syndecan-1 was significantly higher in group NS than in group C (*P* = 0.0009), but no other groups (vs. group NS-A, *P* = 0.329/vs. group NS-V; *P* = 0.999) showed a significant difference compared to C (Fig. 5).

**Fig. 6:**
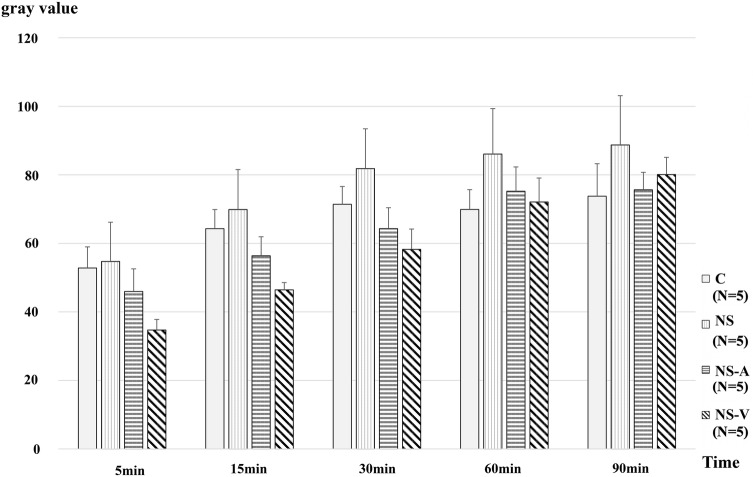
Changes in fluorescence intensity (TMR-DEX40) in the interstitial space. Sequential changes in the fluorescence intensity of TMR at the indicated times in each group administered with TMR-DEX40 after fluid resuscitation with the indicated fluids following blood removal. *N* = 5 per group. *C* untreated control group without blood loss or infusion, *NS* normal saline infusion group, *NS-A* normal saline and albumin infusion group, *NS-V* saline and HES130 infusion group. Values are expressed as the mean ± SE. The data were analyzed by one-way analysis of variance (ANOVA) and the Bonferroni post hoc test. There were no significant differences in TMR-DEX40 leakage between all groups

There were no significant differences in the fluorescence intensity of TMR-DEX40 in the interstitial space among all four groups (Fig. 6, Supplemental Table 2).

**Fig. 7:**
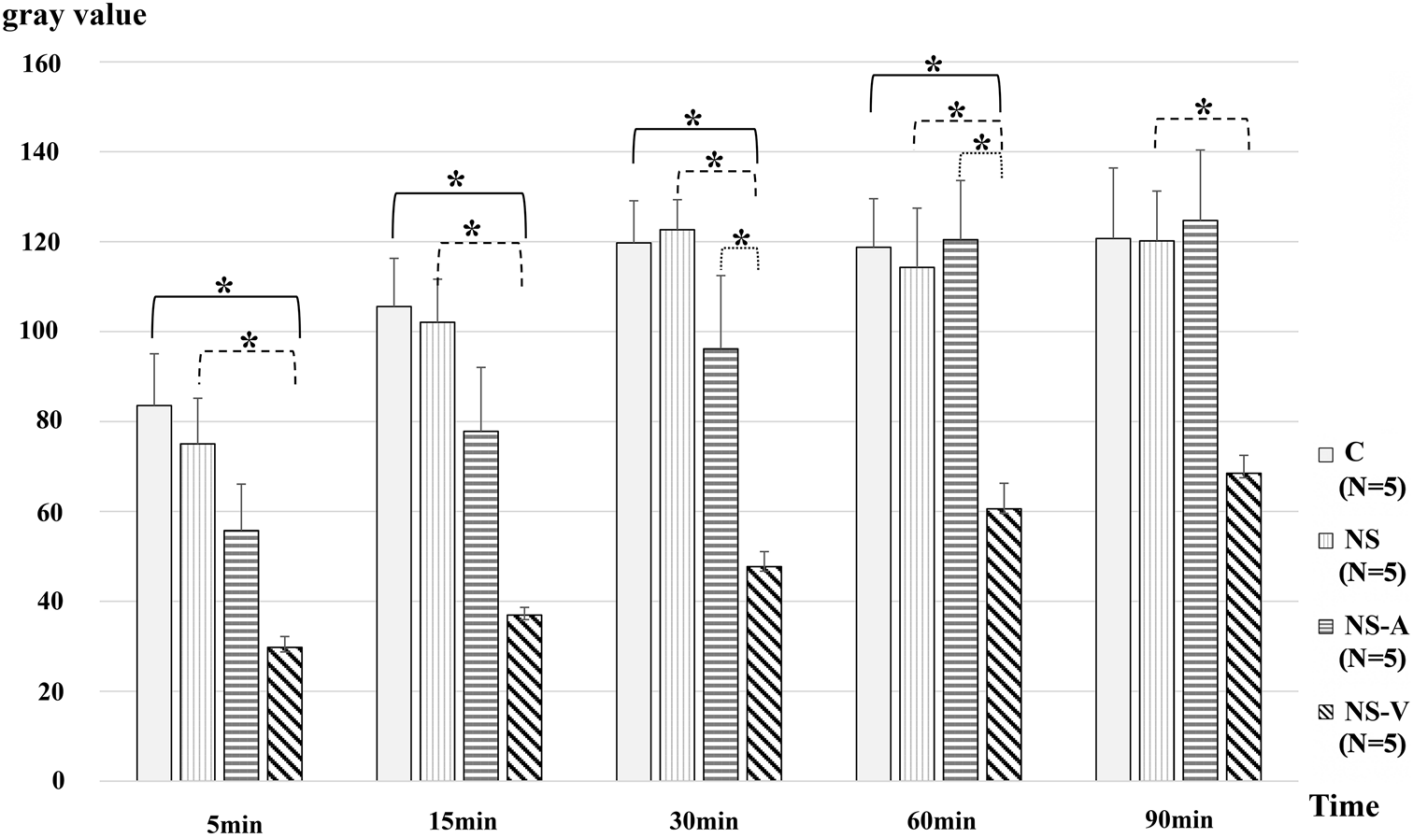
Changes in fluorescence intensity (FITC-HES70) in the interstitial space. Sequential changes in the fluorescence intensity of FITC at the indicated times in each group administered with FITC-HES70 after fluid resuscitation with the indicated fluids after blood removal. Values are expressed as the mean ± SE; *N* = 5 per group. *C* untreated control group without blood loss or infusion; NS, normal saline infusion group, *NS-A* normal saline and albumin infusion group, *NS-V* saline and HES130 infusion group. The data were analyzed by one-way analysis of variance (ANOVA) and the Bonferroni post-hoc test. *P < 0.05,

The FITC-HES70 fluorescence intensity in the interstitial space was significantly suppressed in group NS-V compared to that in group C (*P* < 0.05 at 5, 15, 30, and 60 min), that in group NS (*P* < 0.05 at 5, 15, 30, 60, and 90 min), and that in group NS-A (*P* < 0.05 at 30 and 60 min) (Fig. 7, Supplemental Table 2).

**Fig. 8:**
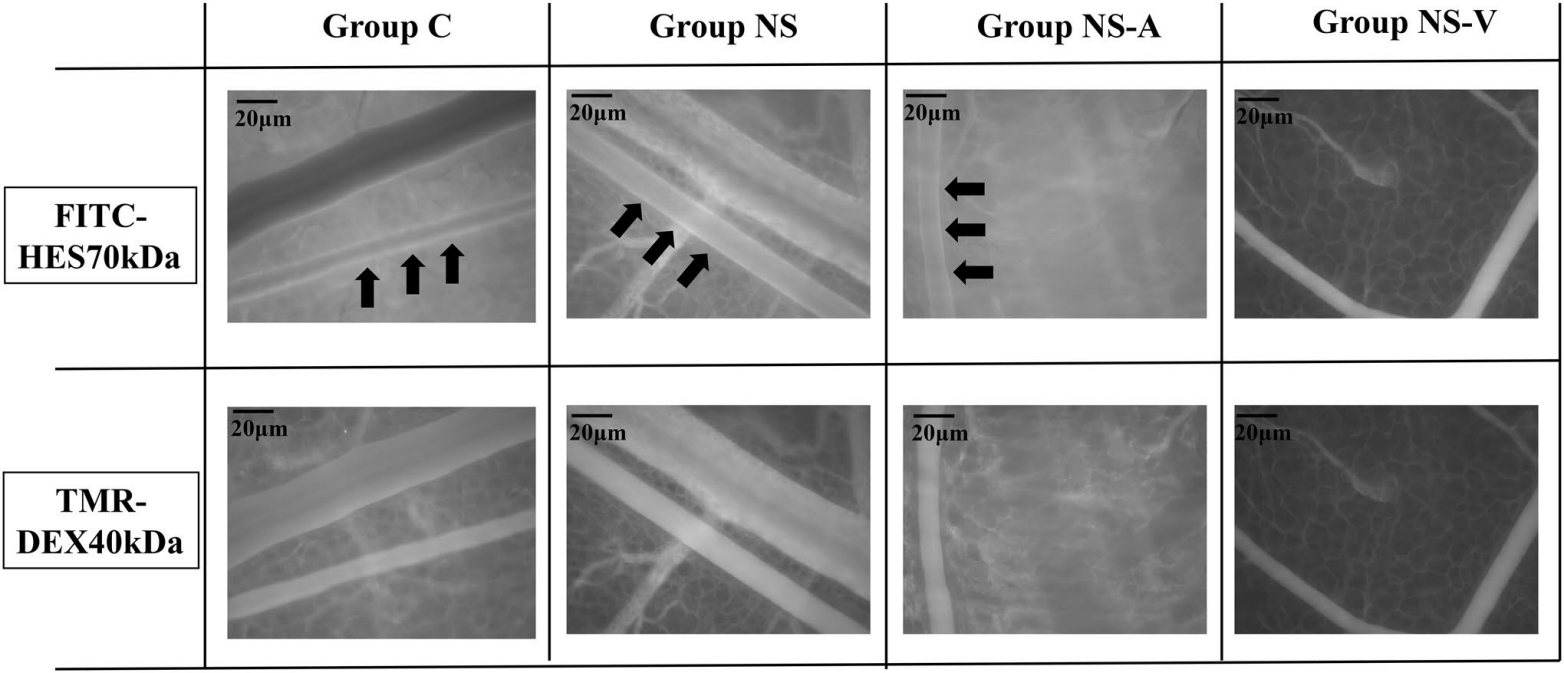
Localization of FITC-HES70 in mouse arteries. Representative images at 60 min after blood removal in each group. Fluorescent TMR-DEX40 uniformly stained the intravascular wall in all groups. FITC-HES70 was localized to the intravascular wall (black arrows) in the untreated control (C) group, normal saline administration (NS) group, and normal saline plus albumin administration (NS-A) group, but was uniformly distributed in the normal saline plus HES130 administration (NS-V) group

The localization of TMR-DEX40 and FITC-HES70 in the blood vessels at 60 min after administration was different between groups. In all groups, TMR-DEX40 was uniformly distributed across the blood vessels. In contrast, FITC-HES70 was localized to the inner wall of the blood vessels in groups C, NS, and NS-A, but was uniformly distributed across the blood vessels in group NS-V (Fig. 8).

Blood gas analysis showed that the pH and lactate values in group NS-V showed the greatest improvement among the treatment groups (Table 1).

**Table 1:** Blood gas analysis parameters

	C (*N* = 10)	NS (*N* = 7)	NS-A (*N* = 8)	NS-V (*N* = 8)
Age (weeks)	13.3 ± 0.79	14.9 ± 0.95	13.5 ± 0.88	13.0 ± 0.79
Weight (g)	26.4 ± 0.96	27.9 ± 1.68	27.4 ± 1.47	27.3 ± 1.55
pH	7.17 ± 0.058	6.97 ± 0.071^*^	7.07 ± 0.052^*^	7.10 ± 0.052
pCO_2_ (mmHg)	83.0 ± 11.73	85.33 ± 10.64	82.74 ± 7.19	83.06 ± 9.80
pO_2_ (mmHg)	46.1 ± 18.23	42.4 ± 9.74	37.6 ± 5.78	40.8 ± 9.55
BE (mEq/l)	1.8 ± 1.48	− 11.9 ± 4.41^*^	− 6.1 ± 3.44^*^	− 3.9 ± 1.85^*^
HCO_3_ (mEq/l)	30.17 ± 1.19	19.82 ± 3.41^*^	23.88 ± 2.64^*^	25.71 ± 1.36^*^
SaO_2_ (mmHg)	62.1 ± 15.53	48.7 ± 12.38	48.1 ± 12.18	54.0 ± 17.53
Lactate (mmol/l)	0.81 ± 0.51	4.3 ± 1.03^*^	2.94 ± 1.34^*^	1.62 ± 1.53
Hct (%)	40.2 ± 0.72	18.0 ± 0.86^*^	15.8 ± 0.80^*^	17.4 ± 0.86^*^
Hb (g/dl)	13.7 ± 1.06	6.1 ± 0.88^*^	4.3 ± 1.44^*^	5.9 ± 0.61^*^

*BE* base excess, *Hct* hematocrit, *Hb* hemoglobin, *C* untreated control group, *NS* group treated with normal saline, *NS-A* group treated with normal saline and albumin, *NS-V* group treated with normal saline and hydroxyethyl starch

Data are shown as the mean ± SD. *P < 0.01 compared to group C

Group NS showed the highest 7-day cumulative mortality among all groups (Supplemental Fig. 3).

## Discussion

The aim of this study was to clarify the effectiveness of HES to treat patients with massive hemorrhages in terms of its protective effect against degradation of the GCX, which plays an important role in regulating ESL hyperpermeability. HES130 protected the GCX layer by maintaining its thickness, and prevented its breakdown as shown by the reduced plasma syndecan-1 concentrations.

In this study, we measured the thickness of the GCX layer in vivo. The GCX in vitro is generally much thinner than that in vivo, because transmission electron micrographs (TEM) of GCX require prior preservation by alcohol dehydration, which removes the water-rich gel layer of the ESL. The observed GCX thickness in TEM is reported to be less than 100 nm. However, Ebong et al. reported that GCX preserved through rapid freezing without dehydration was thicker [21]. Because the GCX layer contains many plasma proteins, including human albumin and coagulation factors, as well as high concentrations of water, and because the human GCX contains approximately 1000 ml plasma at the ESL under normal conditions owing to the presence of a gel layer in the upper GCX layer [22], the thickness of physiologically active GCX in vivo is greater than what has previously been detected in vitro by conventional TEM. Therefore, we obtained intravital images of the GCX using FITC-WGA, which attached to components of the GCX. We measured the thickness of the FITC-WGA-positive layer as a surrogate for GCX thickness. Our measurement of GCX thickness by intravital microscopy showed a thickness of approximately 4 μm. This is considered to be a reliable method of measuring GCX thickness in vivo. We observed that GCX index was significantly lower in only group NS than in group C, and there were no significant differences in group NS-A and NS-V compared to group C. Consistent with the observed results for GCX thickness, the plasma concentration of syndecan-1 was significantly higher in group NS than in group C, indicating that fluid resuscitation using only normal saline leads to GCX damage [11], resulting in a poor prognosis [23–25].

We also demonstrated that the NS-V-treated group showed a significantly lower fluorescence intensity of FITC-HES70 in the interstitial space at every observational time point than did the NS-treated group, suggesting that HES130 suppressed vascular permeability during fluid resuscitation in our blood removal model.

TMR-DEX40 and FITC-HES70 were selected to investigate the changes in vascular permeability for several reasons. GCX has several functions in the endothelium, and the GCX acts as an interface between chemical signals in the blood, biochemical pathways inside cells, and a barrier against chemical and mechanical damage [26]. Among several factors related to vascular permeability, such as the size barrier, electron barrier, substance concentration, and the physiological and electrical force of GCX, we focused on the endothelial size barrier with a “large pores and small pores” system [27]; for this reason, TMR-DEX40 was chosen. Because TMR-DEX40 is slightly larger than the small pores of the barrier, we hypothesized that even slight damage to the barrier would lead to an impairment of the endothelial layer, resulting in the extravascular leakage of TMR-DEX40. However, the results showed no significant differences in TMR-DEX40 leakage between the groups. In addition, TMR-DEX40 was found to gradually leak out of the plasma to the interstitial space over time, even in intact vessels. These results suggest that the conformation of DEX40 might be slightly smaller than we expected. We thus also used FITC-HES70, a different fluorescent tracer, to track the movement of HES in mice with massive hemorrhages.

The leakage of HES70 in the NS-V group was lower than that in the C group. However, there were no significant differences in the leakage of TMR-DEX40 between any groups at every time point. According to a previous study that performed an intravital analysis of vascular permeability in mice using two-photon microscopy [28], FITC-DEX40 gradually moves into interstitial spaces even under normal conditions. Thus, it is understandable that, in this study, the fluorescence intensity of TMR-DEX40 in the interstitial space gradually increased in group C.

The reduced fluorescence intensity of FITC-HES70 in the interstitial space in group NS-V may have been due to the following reason. Unlike DEX40, which is not degraded in plasma, HES70 is rapidly degraded by plasma amylase after infusion [29]. HES70 is broken down into small molecules of less than 40 kDa, which is considered the threshold size for leakage into the interstitial space. This may be why the rapidly degraded FITC-HES70 in our study leaked into the interstitial space in groups C, NS, and NS-A, and why degradation and leakage increased over time. However, this was not observed in group NS-V; although the leakage of HES70 in group NS-V increased over time, it was significantly lower than that in group C at every time point except 90 min. We speculate that HES130 administration enhanced small pore barrier function through its direct adherence to GCX to prevent even the degraded products of 70 kDa HES from passing through the small pore system.

Fluorescence images of arteries in the DSC showed that HES70 was localized to the inner wall of vessels in groups C, NS, and NS-A, but not in group NS-V. The reason was that HES130, which was administered prior to HES70, attached to the GCX and prevented HES70 from getting close to the GCX as well as leaking into the interstitial space. Therefore, HES70 was uniformly present in the vessel lumen. TMR-DEX40 was uniformly distributed across the blood vessels in all groups, suggesting that TMR-DEX40 and FITC-HES70 showed different movements in plasma (Fig. 8). We suggest that HES130 preferentially binds to the surface of endothelial cells to prevent the leakage of HES70, thus protecting endothelial cell function (Supplemental Movies).

Blood gas analysis showed that, compared to group NS, the pH, base excess, HCO_3_, and lactate values were significantly improved in group NS-V. The 7-day survival rate was also substantially higher in group NS-A than in group NS. Although blood pressure was not measured in this study, it can be inferred that the recovery time from shock would be shorter after NS-A and NS-V treatment than after NS treatment. In a clinical setting, HES130 could be expected to have at least the same effect as albumin for fluid therapy in patients with massive bleeding.

Based on the results of our study, fluid resuscitation in patients with massive hemorrhages with only normal saline should be avoided, as this may enhance the damage to endothelial cells. Albumin or colloid solution is a more effective fluid therapy to suppress GCX degradation due to massive acute hemorrhages. We have shown that the infusion of HES130 solution had major advantages, including the localization of HES to the surface of endothelial cells, which may directly preserve endothelial cell function to prevent permeability in a severe clinical state. However, the mechanisms surrounding this must still be clarified.

There are many reports on the effects of HES130 administration in patients with massive hemorrhages; HES130 has been shown to shorten the recovery time from shock [12], improve peripheral circulation and vascular permeability, improve the symptoms of edema in small intestinal mucosa [30], and decrease the concentrations of GCX fragmentation products (syndecan-1, heparanase, and hyaluronic acid) [31]. In addition, in a clinical study of HES130 treatment in trauma patients, an improvement of base excess [32] and lactic acid levels was reported. Rossaint et al. reported that HES130 treatment reduced neutrophil–platelet aggregates, chemokine-induced arrest, and the transmigration of neutrophils; HES130 has also been found to have anti-inflammatory effects [14]. In this study, our results suggested that the infusion of HES130 for massive hemorrhage may be highly beneficial to preserve GCX function. Recently, hypotension [33–35] and restrictive infusion [36] have been recommended as infusion strategies for acute hemorrhage in trauma patients. In that regard, it is possible to reduce the required infusion volume through HES130 administration.

There were several limitations to this study. First, considering the results of recent several large randomized clinical trials where the crystalloid fluid volume was around 1.2-fold higher than the colloid fluid volume [37, 38], the volume of NS infusion in this study was slightly lower than that in these previous reports. Because this study focused on the effect of rapid fluid resuscitation in the early stage of massive hemorrhage, we set the blood withdrawal volume to 1.5 mL, which is considered life-threatening blood loss for a mouse whose whole blood volume is around 2.5 mL. In this severe hypovolemic situation, most resuscitation fluid, which is rapidly infused, would remain in the intravascular space due to context-sensitive effects [39], at least during the early stage of massive hemorrhage. Furthermore, based on the blood volume of a mouse, we considered that rapid fluid resuscitation with volumes 1.5-fold higher than the volume of blood removed can lead to right ventricular failure. Therefore, we considered that an equal volume of NS and HES130 infusion would be ideal for fluid resuscitation in this study.

Second, we did not investigate the effects of HES on renal dysfunction, coagulation impairment, or allergies; these are major potential adverse effects of HES infusion in clinical practice. It is still unclear whether fluid resuscitation with HES at the early stage of massive hemorrhage is crucial for the prognosis of patients, because controlling these adverse effects also contributes to prognosis. Large-scale randomized control studies may be necessary in order to evaluate the usefulness of HES130 infusion in massive hemorrhages in clinical practice. However, the results of this study suggest that the localization of HES to the inner vessel wall could lead to an improved strategy for fluid therapy to cope with hyperpermeability caused by the pathological impairment of endothelial cell function.

In conclusion, during early stages of acute massive hemorrhages, an equal volume of saline fluid resuscitation impaired GCX function, whereas HES130 inhibited the progress of GCX injury and suppressed vascular permeability. The localization of HES to the inner vessel wall suggested that HES might have direct protective mechanisms for the GCX.

## Electronic supplementary material

Below is the link to the electronic supplementary material.

Supplemental movie. Localization of fluorescein-labelled hydroxyethyl starch (FITC-HES130). Micro-circulation at 15 min after administration of FITC-HES130. The inner arteriolar wall was strongly illuminated by the fluorescent dyes, indicating that HES130 was localized at the inner wall of the blood vessels. (MP4 2108 kb)

Supplemental movie. Localization of fluorescein-labelled hydroxyethyl starch (FITC-HES130). Micro-circulation at 60 min after administration of FITC-HES130. (MP4 1840 kb)

Supplemental movie. Localization of fluorescein-labelled hydroxyethyl starch (FITC-HES130). Micro-circulation at 120 min after administration of FITC-HES130. The inner arteriolar wall was still illuminated by the fluorescent dye, even after 120 min. (MP4 815 kb)

Supplemental Table 1. **Average age and weight of all mice relative to each experimental result.** Supplemental Table 1 shows the obtained data from the mice used in our study (fluorescence intensity of TMR-DEX40 and FITC-HES70 in the interstitial space, GCX index, syndecan-1 blood concentration, blood gas analysis, and 7-day cumulative mortality). This table also contains data on the age (weeks) and body weight of all mice used in these experiments. The body weights included the weight of the dorsal skinfold chambers (DSCs; 1.5 g); mice were weighed before the start of experiments. For intravital microscopy experiments with fluorescent dyes, five mice were included in each group. The mice used for these experiments were carefully selected and confirmed to be adequate for the observation of DSCs within the ideal age and weight ranges. To minimize the number of animals, we used mice whose implanted DSCs were not suitable even after waiting a few weeks to observe microcirculation, for the study of syndecan-1 blood concentration, blood gas analysis, and the seven-day cumulative mortality experiments, leading to variations in age and body weight. (PPTX 51 kb)

Supplemental Table 2. **Average fluorescence intensity in the interstitial space at all time points**. Supplemental Table 2 shows the fluorescence intensity of TMR-DEX40 and FITC-HES70 as an index of leakage into the peripheral area in the DSCs in all groups. TMR-DEX40 leakage was measured by examining the average fluorescence intensity over the interstitial space (30 × 30 μm) at 5, 15, 30, 60, and 90 min. ImageJ software was used for the analysis of fluorescent images. The software assigned an integer value to the brightness of the fluorescence signal using an 8-bit gray scale (range, 0–255) in each region of interest. (PPTX 45 kb)

Supplemental Fig. 1. **Dorsal skinfold chamber.** A dorsal skin chamber (DSC) was used to visualize the microvasculatures. Briefly, the DSC chamber frame was constructed from poly-acetal resin, as in our previous study [15]. Two frames were surgically implanted, so that the extended double-layer of the dorsal skin was sandwiched. A coverslip was then fixed with a retaining ring. During the surgical procedure, mice were anesthetized by subcutaneous injection of a mixture of ketamine (90 mg/kg body weight) and xylazine (10 mg/kg body weight). Mice were allowed to acclimatize for at least 1 week before microscopic observations to avoid any inflammatory effects due to surgery. (PPTX 57 kb)

Supplemental Fig. 2. **Measurement of GCX thickness index**. After inducing acute hemorrhage in the four groups as described above, the mice were left to stabilize for about 5 min and then injected with FITC-WGA. After 30 min, three fluorescent images were obtained in each chamber. The artery walls were clearly illuminated by FITC-WGA lectin. Fluorescence images of FITC-WGA-stained regions were analyzed using ImageJ software. Three arteries of approximately 20 μm in diameter were selected in each image, and the fluorescence intensity was measured across three lines perpendicular to the artery walls in each chamber to compare changes in the GCX thickness between groups (Supplemental Fig. 2a). GCX thickness indexes were defined as follows: A, peak of fluorescence intensity; B, halfway point between peak and baseline; C, baseline; and D, thickness of FITC-WGA positive layer, GCX thickness index (Supplemental Fig. 2b). The GCX thickness index was considered to be approximately the same as the thickness of the GCX layer. (PPTX 398 kb)

Supplemental Fig. 3. **Seven-day cumulative mortality rate**. The seven-day cumulative mortality was determined in each group of mice after surgery by removing the blood withdrawal catheter without fluorochrome administration. C, untreated control group without blood loss or infusion; NS, normal saline infusion group; NS-A, normal saline and albumin infusion group; NS-V, saline and HES130 infusion group. The NS group showed the highest 7-day cumulative mortality among all groups. (PPTX 185 kb)
