# Supplementary material for: The protective effect of hydroxyethyl starch solution on the glycocalyx layer in an acute hemorrhage mouse model
Source: J Anesth. 2019 Oct 15;34(1):36–46. doi: 10.1007/s00540-019-02692-8 (PMC6992552; doi:10.1007/s00540-019-02692-8)
Supplement: Supplementary file 4 — Supplemental Table 1. Average age and weight of all mice relative to each experimental result. Supplemental Table 1 shows the obtained data from the mice used in our study (fluorescence intensity of TMR-DEX40 and FITC-HES70 in the interstitial space, GCX index, syndecan-1 blood concentration, blood gas analysis, and 7-day cumulative mortality). This table also contains data on the age (weeks) and body weight of all mice used in these experiments. The body weights included the weight of the dorsal skinfold chambers (DSCs; 1.5 g); mice were weighed before the start of experiments. For intravital microscopy experiments with fluorescent dyes, five mice were included in each group. The mice used for these experiments were carefully selected and confirmed to be adequate for the observation of DSCs within the ideal age and weight ranges. To minimize the number of animals, we used mice whose implanted DSCs were not suitable even after waiting a few weeks to observe microcirculation, for the study of syndecan-1 blood concentration, blood gas analysis, and the seven-day cumulative mortality experiments, leading to variations in age and body weight. (PPTX 51 kb) [file 540_2019_2692_MOESM4_ESM.pptx]

## Slide 1
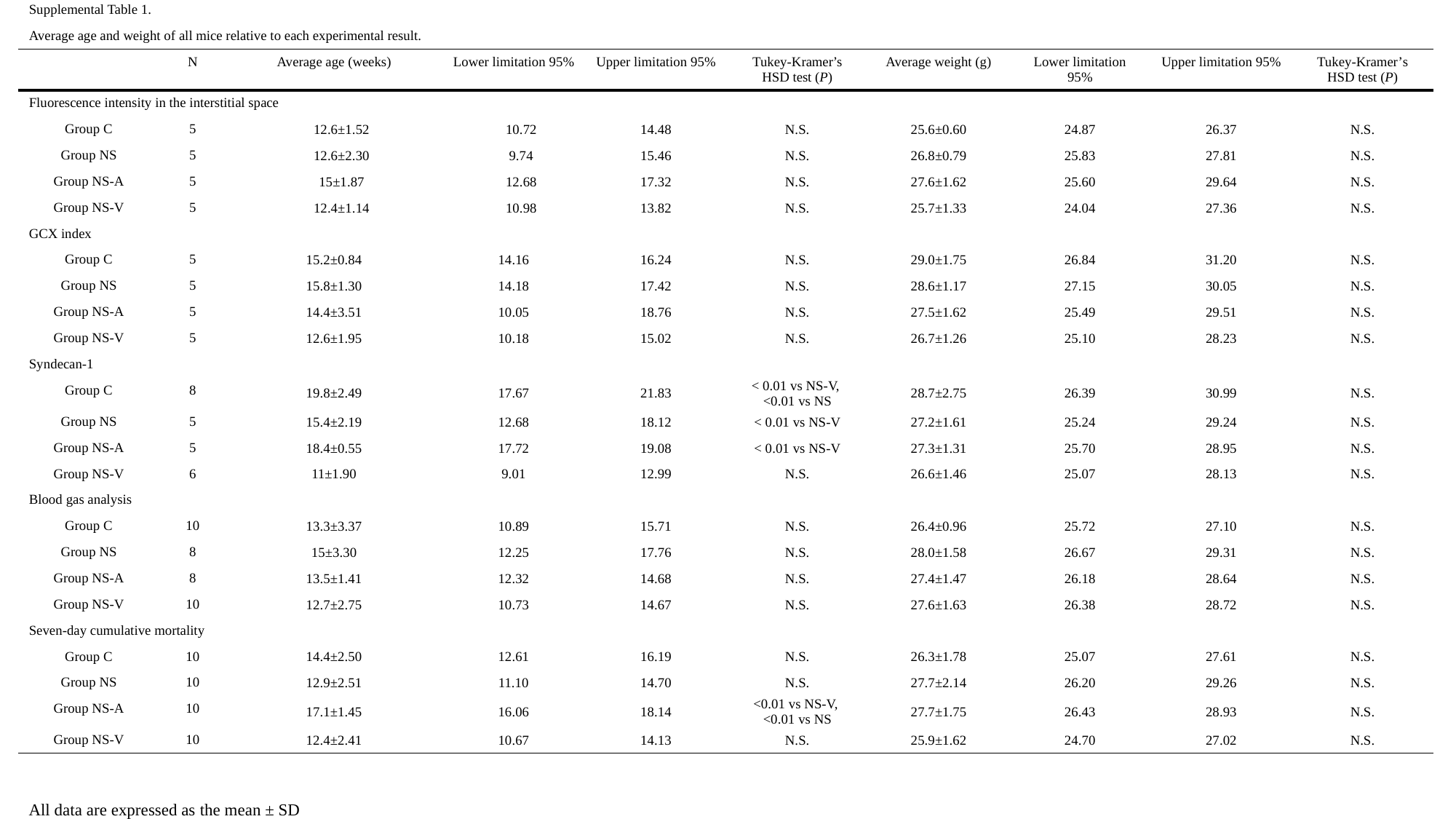

| Supplemental Table 1. | | | | | | | | | | |
| --- | --- | --- | --- | --- | --- | --- | --- | --- | --- | --- |
| Average age and weight of all mice relative to each experimental result. | | | | | | | | | | |
| | N | Average age (weeks) | Lower limitation 95% | | Upper limitation 95% | Tukey-Kramer’s HSD test (P) | Average weight (g) | Lower limitation 95% | Upper limitation 95% | Tukey-Kramer’s HSD test (P) |
| Fluorescence intensity in the interstitial space | | | | | | | | | | |
| Group C | 5 | 12.6±1.52 | 10.72 | 10.72 | 14.48 | N.S. | 25.6±0.60 | 24.87 | 26.37 | N.S. |
| Group NS | 5 | 12.6±2.30 | 9.74 | 9.74 | 15.46 | N.S. | 26.8±0.79 | 25.83 | 27.81 | N.S. |
| Group NS-A | 5 | 15±1.87 | 12.68 | 12.68 | 17.32 | N.S. | 27.6±1.62 | 25.60 | 29.64 | N.S. |
| Group NS-V | 5 | 12.4±1.14 | 10.98 | 10.98 | 13.82 | N.S. | 25.7±1.33 | 24.04 | 27.36 | N.S. |
| GCX index | | | | | | | | | | |
| Group C | 5 | 15.2±0.84 | 14.16 | | 16.24 | N.S. | 29.0±1.75 | 26.84 | 31.20 | N.S. |
| Group NS | 5 | 15.8±1.30 | 14.18 | | 17.42 | N.S. | 28.6±1.17 | 27.15 | 30.05 | N.S. |
| Group NS-A | 5 | 14.4±3.51 | 10.05 | | 18.76 | N.S. | 27.5±1.62 | 25.49 | 29.51 | N.S. |
| Group NS-V | 5 | 12.6±1.95 | 10.18 | | 15.02 | N.S. | 26.7±1.26 | 25.10 | 28.23 | N.S. |
| Syndecan-1 | | | | | | | | | | |
| Group C | 8 | 19.8±2.49 | 17.67 | | 21.83 | < 0.01 vs NS-V, <0.01 vs NS | 28.7±2.75 | 26.39 | 30.99 | N.S. |
| Group NS | 5 | 15.4±2.19 | 12.68 | | 18.12 | < 0.01 vs NS-V | 27.2±1.61 | 25.24 | 29.24 | N.S. |
| Group NS-A | 5 | 18.4±0.55 | 17.72 | | 19.08 | < 0.01 vs NS-V | 27.3±1.31 | 25.70 | 28.95 | N.S. |
| Group NS-V | 6 | 11±1.90 | 9.01 | | 12.99 | N.S. | 26.6±1.46 | 25.07 | 28.13 | N.S. |
| Blood gas analysis | | | | | | | | | | |
| Group C | 10 | 13.3±3.37 | 10.89 | | 15.71 | N.S. | 26.4±0.96 | 25.72 | 27.10 | N.S. |
| Group NS | 8 | 15±3.30 | 12.25 | | 17.76 | N.S. | 28.0±1.58 | 26.67 | 29.31 | N.S. |
| Group NS-A | 8 | 13.5±1.41 | 12.32 | | 14.68 | N.S. | 27.4±1.47 | 26.18 | 28.64 | N.S. |
| Group NS-V | 10 | 12.7±2.75 | 10.73 | | 14.67 | N.S. | 27.6±1.63 | 26.38 | 28.72 | N.S. |
| Seven-day cumulative mortality | | | | | | | | | | |
| Group C | 10 | 14.4±2.50 | 12.61 | | 16.19 | N.S. | 26.3±1.78 | 25.07 | 27.61 | N.S. |
| Group NS | 10 | 12.9±2.51 | 11.10 | | 14.70 | N.S. | 27.7±2.14 | 26.20 | 29.26 | N.S. |
| Group NS-A | 10 | 17.1±1.45 | 16.06 | | 18.14 | <0.01 vs NS-V, <0.01 vs NS | 27.7±1.75 | 26.43 | 28.93 | N.S. |
| Group NS-V | 10 | 12.4±2.41 | 10.67 | | 14.13 | N.S. | 25.9±1.62 | 24.70 | 27.02 | N.S. |
All data are expressed as the mean ± SD
