# Supplementary material for: The protective effect of hydroxyethyl starch solution on the glycocalyx layer in an acute hemorrhage mouse model
Source: J Anesth. 2019 Oct 15;34(1):36–46. doi: 10.1007/s00540-019-02692-8 (PMC6992552; doi:10.1007/s00540-019-02692-8)
Supplement: Supplementary file 5 — Supplemental Table 2. Average fluorescence intensity in the interstitial space at all time points. Supplemental Table 2 shows the fluorescence intensity of TMR-DEX40 and FITC-HES70 as an index of leakage into the peripheral area in the DSCs in all groups. TMR-DEX40 leakage was measured by examining the average fluorescence intensity over the interstitial space (30 × 30 μm) at 5, 15, 30, 60, and 90 min. ImageJ software was used for the analysis of fluorescent images. The software assigned an integer value to the brightness of the fluorescence signal using an 8-bit gray scale (range, 0–255) in each region of interest. (PPTX 45 kb) [file 540_2019_2692_MOESM5_ESM.pptx]

## Slide 1
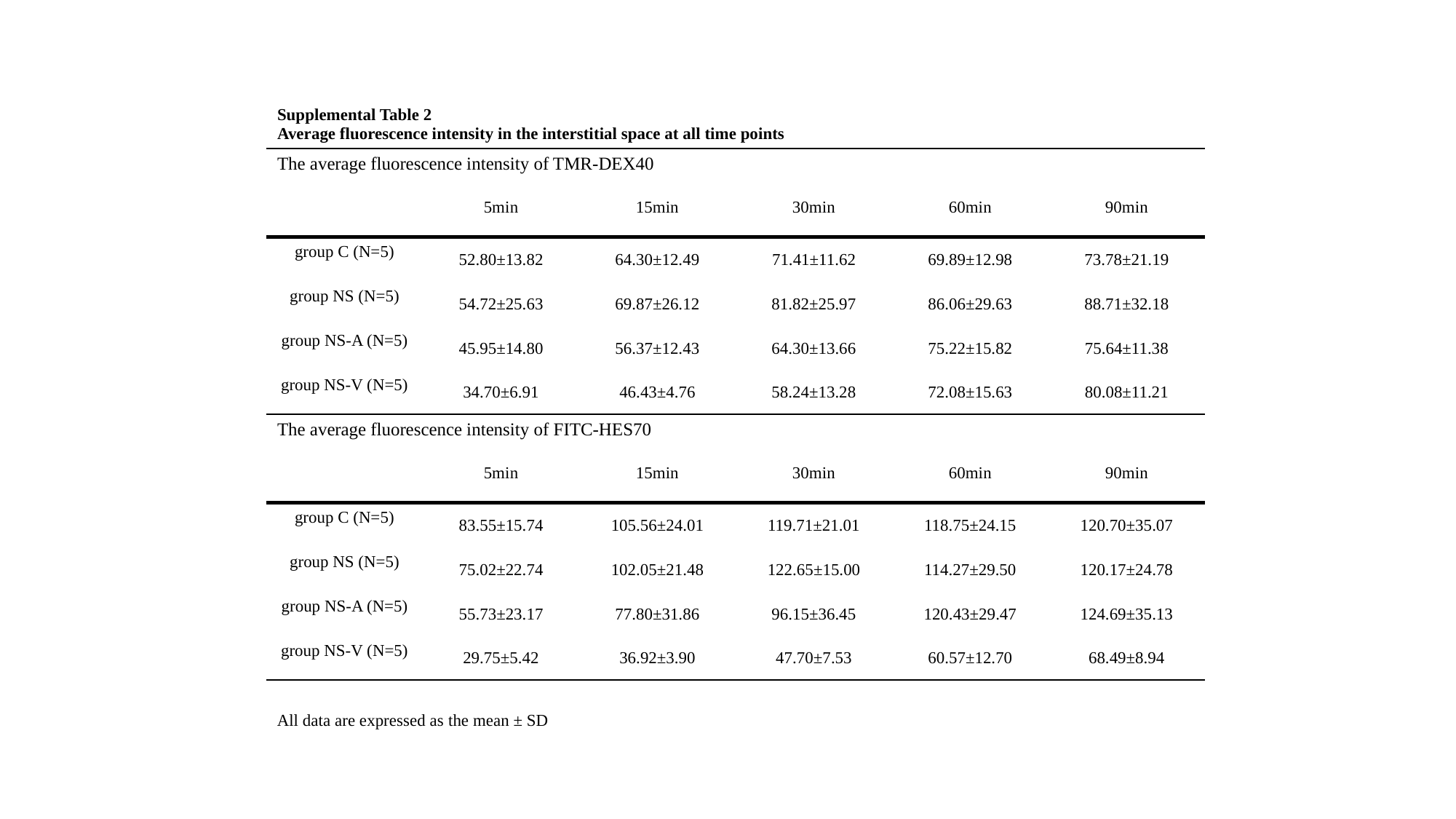

| Supplemental Table 2 Average fluorescence intensity in the interstitial space at all time points | | | | | |
| --- | --- | --- | --- | --- | --- |
| The average fluorescence intensity of TMR-DEX40 | | | | | |
| | 5min | 15min | 30min | 60min | 90min |
| group C (N=5) | 52.80±13.82 | 64.30±12.49 | 71.41±11.62 | 69.89±12.98 | 73.78±21.19 |
| group NS (N=5) | 54.72±25.63 | 69.87±26.12 | 81.82±25.97 | 86.06±29.63 | 88.71±32.18 |
| group NS-A (N=5) | 45.95±14.80 | 56.37±12.43 | 64.30±13.66 | 75.22±15.82 | 75.64±11.38 |
| group NS-V (N=5) | 34.70±6.91 | 46.43±4.76 | 58.24±13.28 | 72.08±15.63 | 80.08±11.21 |
| The average fluorescence intensity of FITC-HES70 | | | | | |
| | 5min | 15min | 30min | 60min | 90min |
| group C (N=5) | 83.55±15.74 | 105.56±24.01 | 119.71±21.01 | 118.75±24.15 | 120.70±35.07 |
| group NS (N=5) | 75.02±22.74 | 102.05±21.48 | 122.65±15.00 | 114.27±29.50 | 120.17±24.78 |
| group NS-A (N=5) | 55.73±23.17 | 77.80±31.86 | 96.15±36.45 | 120.43±29.47 | 124.69±35.13 |
| group NS-V (N=5) | 29.75±5.42 | 36.92±3.90 | 47.70±7.53 | 60.57±12.70 | 68.49±8.94 |
All data are expressed as the mean ± SD
