# Supplementary figures and images for: The protective effect of hydroxyethyl starch solution on the glycocalyx layer in an acute hemorrhage mouse model
Source: J Anesth. 2019 Oct 15;34(1):36–46. doi: 10.1007/s00540-019-02692-8 (PMC6992552; doi:10.1007/s00540-019-02692-8)

## Slide 1
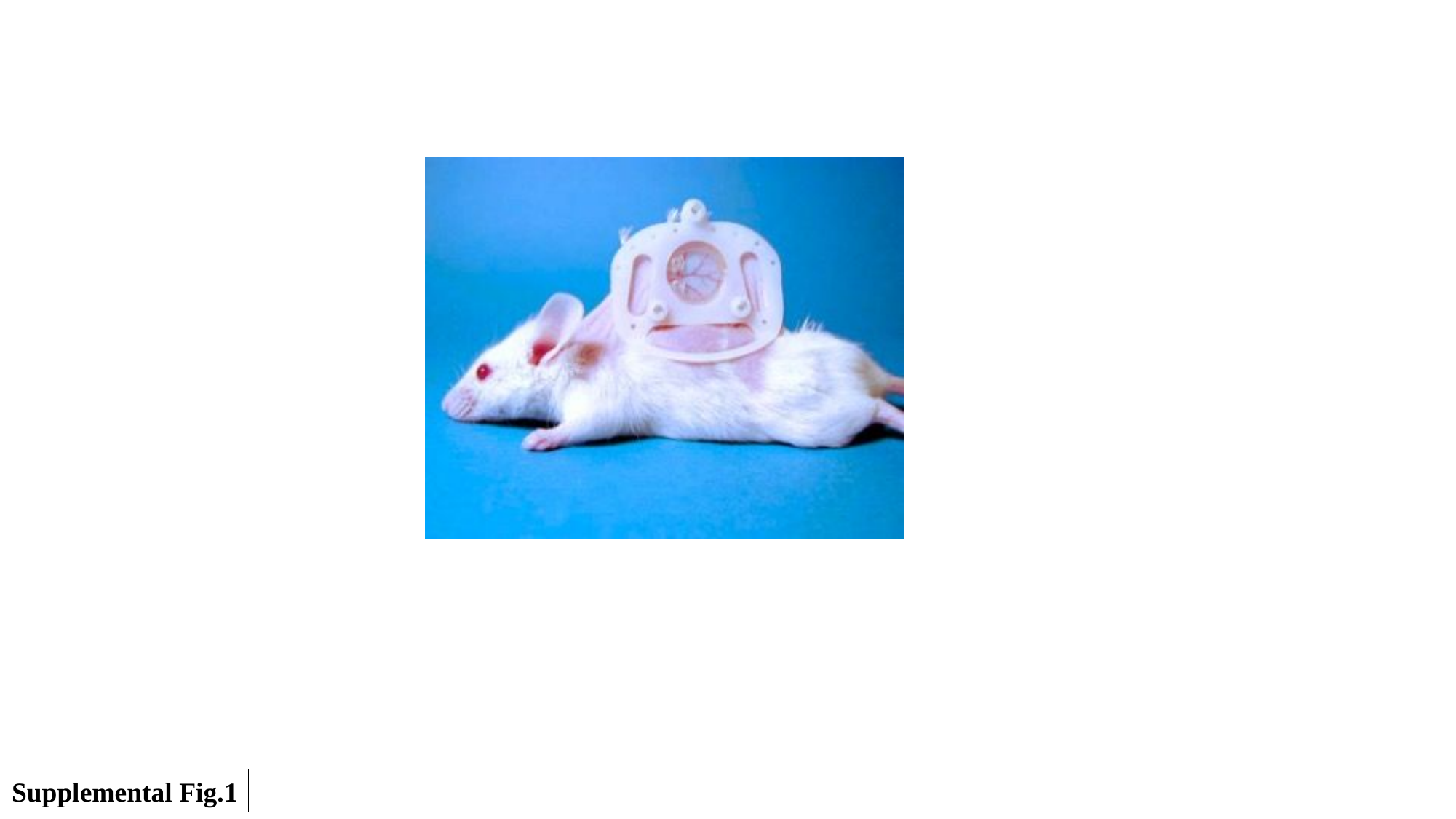

Supplemental Fig.1

Supplement: Supplementary file 6 — Supplemental Fig. 1. Dorsal skinfold chamber. A dorsal skin chamber (DSC) was used to visualize the microvasculatures. Briefly, the DSC chamber frame was constructed from poly-acetal resin, as in our previous study [15]. Two frames were surgically implanted, so that the extended double-layer of the dorsal skin was sandwiched. A coverslip was then fixed with a retaining ring. During the surgical procedure, mice were anesthetized by subcutaneous injection of a mixture of ketamine (90 mg/kg body weight) and xylazine (10 mg/kg body weight). Mice were allowed to acclimatize for at least 1 week before microscopic observations to avoid any inflammatory effects due to surgery. (PPTX 57 kb) [file 540_2019_2692_MOESM6_ESM.pptx]

## Slide 1
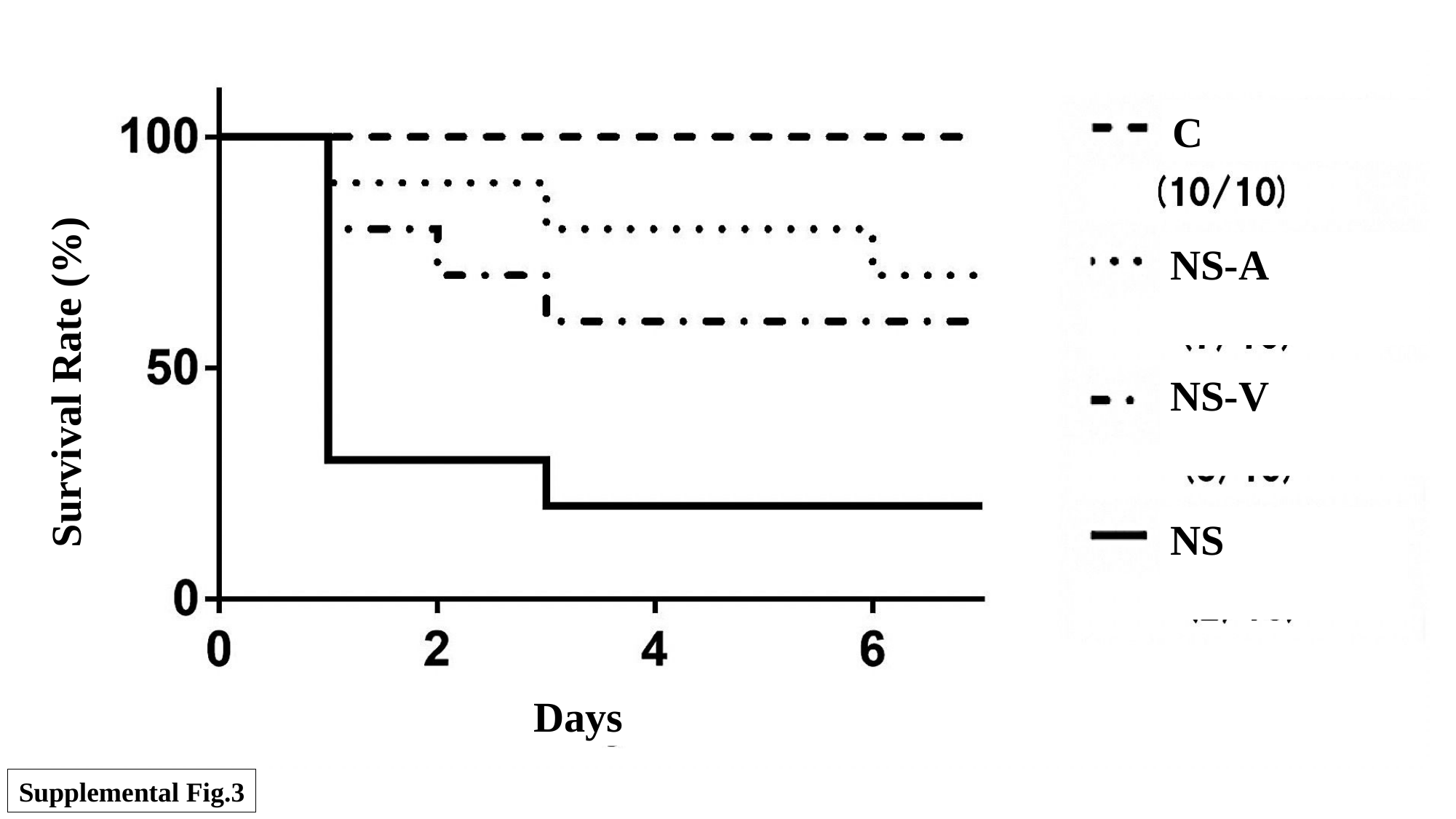

Survival Rate (%)
C
NS-A
NS-V
NS
Days
Supplemental Fig.3

Supplement: Supplementary file 8 — Supplemental Fig. 3. Seven-day cumulative mortality rate. The seven-day cumulative mortality was determined in each group of mice after surgery by removing the blood withdrawal catheter without fluorochrome administration. C, untreated control group without blood loss or infusion; NS, normal saline infusion group; NS-A, normal saline and albumin infusion group; NS-V, saline and HES130 infusion group. The NS group showed the highest 7-day cumulative mortality among all groups. (PPTX 185 kb) [file 540_2019_2692_MOESM8_ESM.pptx]
