# Supplementary material for: The protective effect of hydroxyethyl starch solution on the glycocalyx layer in an acute hemorrhage mouse model
Source: J Anesth. 2019 Oct 15;34(1):36–46. doi: 10.1007/s00540-019-02692-8 (PMC6992552; doi:10.1007/s00540-019-02692-8)
Supplement: Supplementary file 7 — Supplemental Fig. 2. Measurement of GCX thickness index. After inducing acute hemorrhage in the four groups as described above, the mice were left to stabilize for about 5 min and then injected with FITC-WGA. After 30 min, three fluorescent images were obtained in each chamber. The artery walls were clearly illuminated by FITC-WGA lectin. Fluorescence images of FITC-WGA-stained regions were analyzed using ImageJ software. Three arteries of approximately 20 μm in diameter were selected in each image, and the fluorescence intensity was measured across three lines perpendicular to the artery walls in each chamber to compare changes in the GCX thickness between groups (Supplemental Fig. 2a). GCX thickness indexes were defined as follows: A, peak of fluorescence intensity; B, halfway point between peak and baseline; C, baseline; and D, thickness of FITC-WGA positive layer, GCX thickness index (Supplemental Fig. 2b). The GCX thickness index was considered to be approximately the same as the thickness of the GCX layer. (PPTX 398 kb) [file 540_2019_2692_MOESM7_ESM.pptx]

## Slide 1
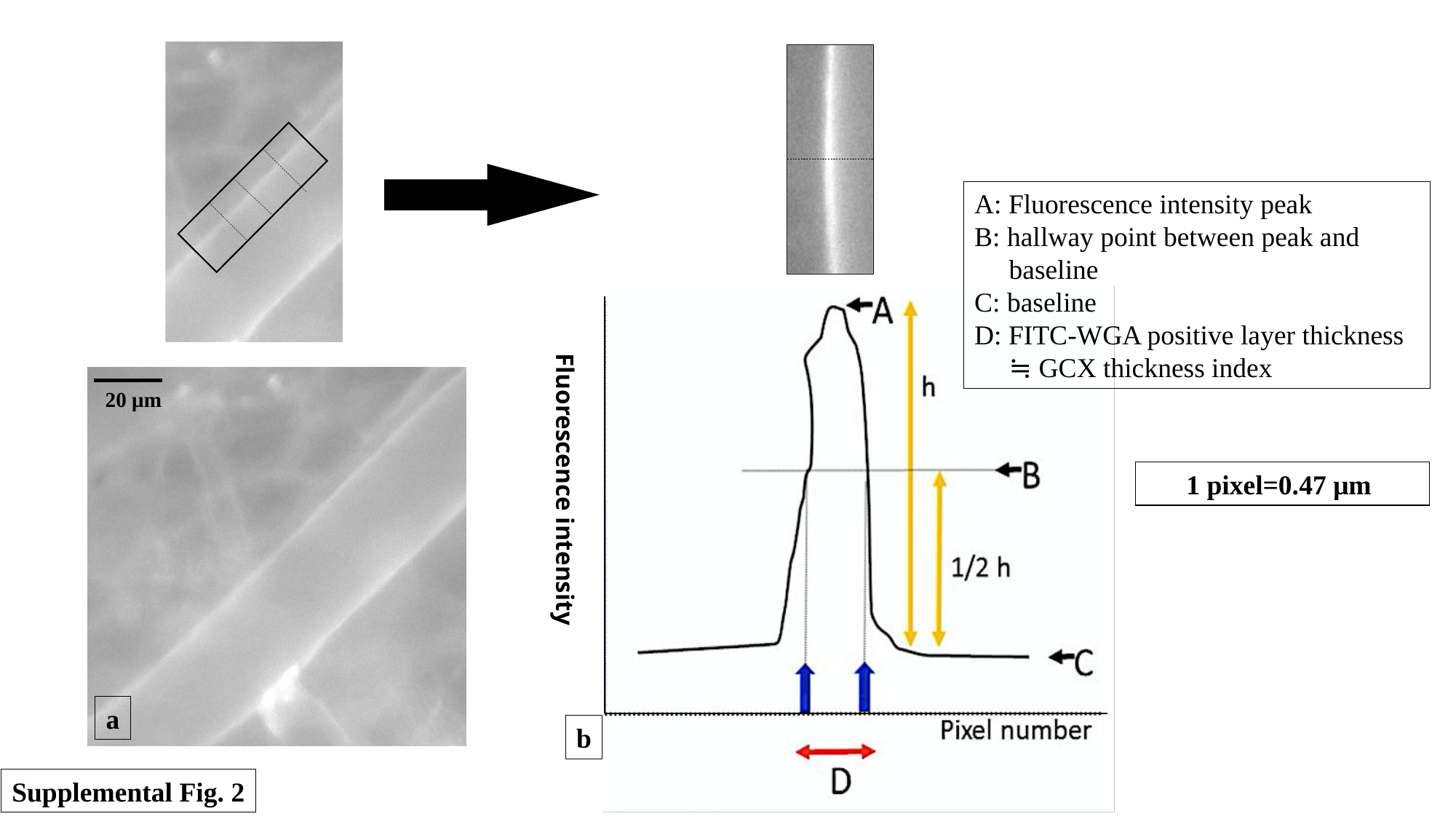

A: Fluorescence intensity peak
B: hallway point between peak and
 baseline
C: baseline
D: FITC-WGA positive layer thickness
 ≒ GCX thickness index
Fluorescence intensity
1 pixel=0.47 μm
20 μm
a
b
Supplemental Fig. 2
